# Dietary fat intake and endometrial cancer risk: dose-response meta-analysis of epidemiological studies

**DOI:** 10.1038/srep16693

**Published:** 2015-11-16

**Authors:** Luo Jiang, Rui Hou, Ting-Ting Gong, Qi-Jun Wu

**Affiliations:** 1 Department of Ultrasound, Shengjing Hospital of China Medical University, Shenyang, China; 2 Department of Obstetrics and Gynecology, Shengjing Hospital of China Medical University, Shenyang, China; 3 Department of Clinical Epidemiology, Shengjing Hospital of China Medical University, Shenyang, China

## Abstract

Epidemiological studies have provided controversial evidence of the association between dietary fat intake and endometrial cancer (EC) risk. To address this inconsistency, we conducted this dose-response meta-analysis by total dietary fat intake, based on epidemiological studies published up to the end of June 2015 identified from PubMed, EMBASE and Web of Science. Two authors (RH and Q-JW) independently performed the eligibility evaluation and data extraction. All differences were resolved by discussion with the third investigator (LJ). Random-effects models were used to estimate summary relative risks (RRs) and 95% confidence intervals (CIs). Overall, the search yielded 16 studies (6 cohort and 10 case-control studies) that involved a total of 7556 EC cases and 563,781 non-cases. The summary RR for EC for each 30g/day increment intake was 0.98 (95%CI = 0.95–1.001; *I*^2^ = 0%; n = 11) for total dietary fat. Non-significant results were observed in plant-based fat (summary RR = 1.05, 95%CI = 0.94–1.18; *I*^2^ = 0%; n = 5) and animal-based fat (summary RR = 1.17, 95%CI = 0.92–1.36; *I*^2^ = 85.0%; n = 6). Additionally, the null associations were observed in almost all the subgroup and sensitivity analyses. In conclusion, findings of the present meta-analysis suggested a lack of association between total dietary fat intake and EC risk. Further studies, especially prospective designed studies are warranted to confirm our findings.

Endometrial cancer (EC) is the fifth most common cancer among women worldwide in 2012; accounting for approximately 0.32 million newly diagnosed cases^1^. Previous studies have suggested that obesity, reproductive factors (e.g., parity, age at menarche), and use of exogenous hormones (e.g., estrogen hormonal replacement therapy, oral contraceptives) were the established risk factors for this disease^2^. However, compared with Africa and South Asia, higher EC incidence rates were observed in North America and Europe, which could not be totally attributed to these aforementioned risk factors^1^. Since diet might be an important difference of lifestyle of these countries, dietary factors have been hypothesized to play roles in the development of EC^2^.

Experimental studies have indicated that several components of diet, including dietary fat intake was involving in the development of EC by modulating the production, metabolism, and excretion of endogenous hormones^3^,^4^,^5^,^6^. However, the epidemiological evidence has still been controversial^6^,^7^,^8^,^9^,^10^,^11^,^12^,^13^,^14^,^15^,^16^,^17^,^18^,^19^,^20^. In 2007, a meta-analysis including 8 studies (one cohort and 7 case-control studies) showed a relative risk (RR) of 1.72 (95% confidence interval (CI) = 1.28–2.32, *I*^2^ = 48.8%, *P* for heterogeneity = 0.07) for the highest compared with the lowest intakes of total dietary fat. However, these included studies reported the aforementioned results with mixed units of dietary fat intake, such as grams/day or % calories from fat^21^. Subsequently, the continuous update project of World Cancer Research Fund and American Institute for Cancer Research (WCRF/AICR) including studies up to December 2012 reported the summary RR per 10 grams of total fat intake per day was 1.00 (95%CI = 0.96–1.04; *I*^2^ = 68.7%, *P* for heterogeneity = 0.04) only based on three prospective studies^22^. Recently, the findings from one of the largest population-based cohort studies, the European Prospective Investigation into Cancer and Nutrition (EPIC) demonstrated that total dietary fat intake was inversely associated with EC risk^7^. In contrast, the Nurses’ Health Studies (NHS/NHSII) updated their evidence but found that no association between total dietary fat intake and EC risk^7^. On the other hand, the relationships between different fat source (plant-based *versus* animal based) intake and EC risk remains inconsistent and elusive which were not summarized in the continuous update project of WCRF/AICR. Additionally, to our knowledge, a comprehensive assessment of the relationship between the different source of fat intake and EC risk has not been reported. Therefore, we carried out this update meta-analysis of epidemiological studies to systematically and quantitatively assess the evidence of total dietary fat intake with EC risk.

## Results

### Search Results, Study Characteristics, and Quality Assessment

**Figure 1:**
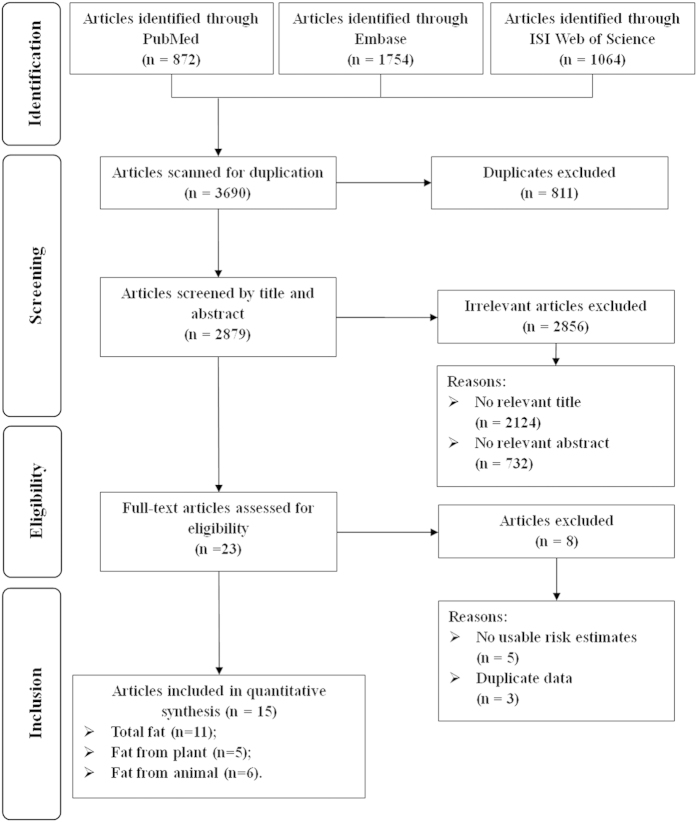
Flow-chart of study selection.

Figure 1 presented the detailed procedures of the article search and screening. Briefly, the search strategy retrieved 3690 unique articles: 872 from PubMed, 1754 from EMBASE, and 1064 from Web of Science. Of these, 3667 articles were excluded after the first screening based on abstracts or titles, leaving 23 articles for full-text review. Among them, eight articles were further excluded due to (i) no usable risk estimates or 95% CIs were reported; and (ii) study population duplication. Overall, a total of 15 articles (16 studies) were included in the present meta-analysis^6^,^7^,^8^,^9^,^10^,^11^,^12^,^13^,^14^,^15^,^16^,^17^,^18^,^19^,^20^.

Characteristics of the 16 selected studies are shown in Table 1. These studies were published between 1993 and 2015 and involved a total of 7556 EC cases and 563,781 non-cases. There were 6 cohort and 10 case-control studies. Of the 6 cohort studies, four were conducted in North America and two in Europe. Of the 10 case-control studies, six were conducted in North America; two were each conducted in Europe and China, respectively. Control subjects were drawn from the general population in 5 studies, hospitals in 5 studies. Age adjusted risk estimates could be determined for all studies. Risk measures were also adjusted for body mass index (14 studies), parity (14 studies), total energy intake (13 studies), oral contraceptive use (11 studies), cigarette smoking (11 studies), menopausal status (11 studies), and hormone replacement therapy (12 studies).

The information of study quality assessment is demonstrated in Tables 2 and 3. Briefly, for the category of “control for important factor or additional factor”, all cohort studies adjusted for more than two potential confounders in their primary analyses except for two^13^,^18^. For the category of “follow-up long enough for outcomes to occur”, all cohort studies were assigned a score except two studies^15^,^18^ because the mean follow-up period of these two studies was less than 10 years. For the category of “using a energy-adjusted model”, two studies^6^,^13^ failed to carry out it in their analysis. (Table 2). Furthermore, for the category of “selection of control subjects”, five case-control studies^9^,^10^,^12^,^17^,^19^ were not assigned a score because the controls of their study were not population-based but hospital-based; For the category of “control for important factor or additional factor”, all case-control studies were assigned two scores except two^17^,^20^; For the category of “exposure assessment”, six case-control studies^8^,^10^,^11^,^12^,^14^,^16^ were assigned a score because their FFQs were validated. Three case-control studies^10^,^16^,^17^ were assigned a score because there was no difference of response rate between cases and controls. Six case-control studies^8^,^10^,^11^,^12^,^14^,^19^ were assigned a score because they presented or considered energy-adjusted model in their primary analyses, respectively (Table 3).

### Dose-response analysis of total dietary fat intake

**Figure 2:**
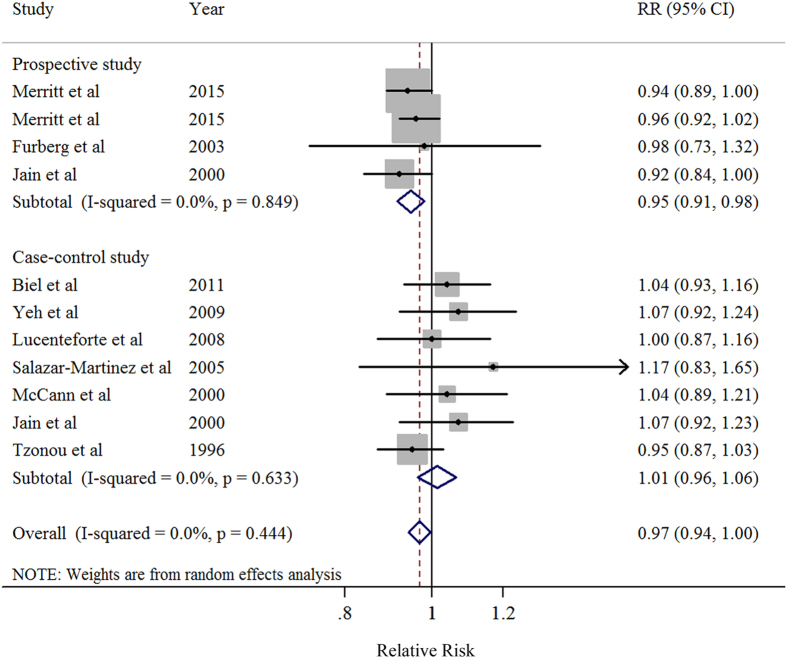
Forest plots (random effect model) of meta-analysis on the relationship between total dietary fat intake and endometrial cancer risk by study design. Squares indicate study-specific risk estimates (size of the square reflects the study-specific statistical weight); horizontal lines indicate 95% CIs; diamond indicates the summary relative risk with its 95% CI. RR: relative risk.

**Figure 3:**
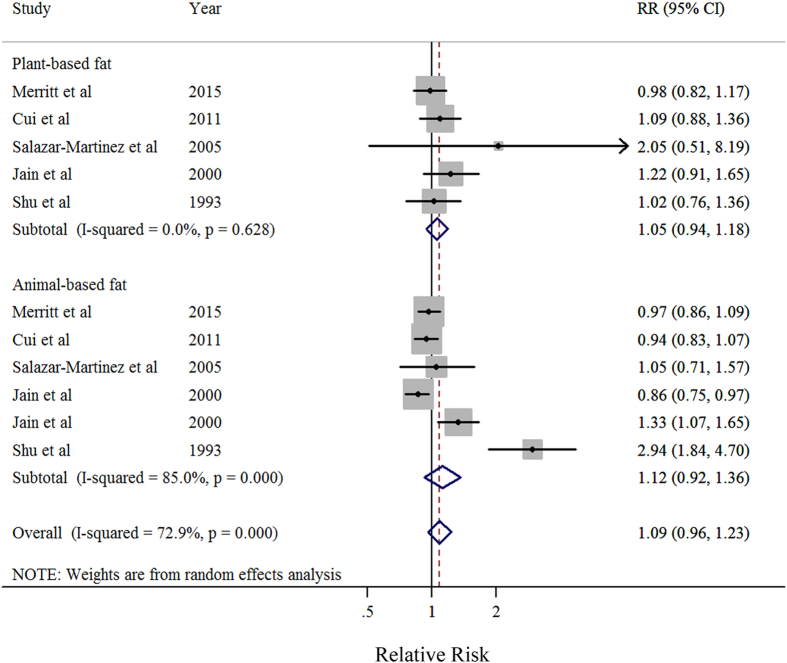
Forest plots (random effect model) of meta-analysis on the relationship between total dietary fat intake and endometrial cancer risk by the source of fat. Squares indicate study-specific risk estimates (size of the square reflects the study-specific statistical weight); horizontal lines indicate 95% CIs; diamond indicates the summary relative risk with its 95% CI. RR: relative risk.

Eleven studies^7^,^8^,^9^,^10^,^12^,^13^,^14^,^15^,^16^,^17^ were included in the dose-response meta-analysis of total dietary fat intake and EC risk (Table 4). The summary RR for a 30g/day increase in total fat intake was 0.97 (95%CI = 0.94–1.001), without heterogeneity (*I*^2^ = 0%, *P* for heterogeneity = 0.44) (Fig. 2). No evidence of a potential nonlinear aforementioned association was observed (*P* for nonlinearity = 0.87). Non-significant results were observed in plant-based fat (RR = 1.05, 95%CI = 0.94–1.18, *I*^2^ = 0%) and animal-based fat (RR = 1.17, 95%CI = 0.92–1.36, *I*^2^ = 85%) (Table 4 and Fig. 3). There was no indication of publication bias by visual inspection of the funnel plot as well as by Egger’s test (*P* for bias = 0.16).

### Subgroup and sensitivity analyses

Although the summary results of cohort studies and studies from Europe showed statistical significance when we carried out the subgroup analyses stratified by study design and geographic location, only three and four studies were included in these analyses which might be partly attributed to chance finding. Furthermore, the non-significant associations between total dietary fat intake and EC risk were observed in almost all the subgroup analyses stratified by number of EC cases per study, whether using the validated FFQ to collect dietary information or energy-adjusted model to analyze the association between focused exposure and outcome, and whether adjustment for potential confounders (Table 4). Additionally, there is no evidence of significant heterogeneity between subgroups with meta-regression analyses.

In a sensitivity analysis of total dietary fat intake and EC risk, we sequentially removed one study at a time and re-analyzed the data. The 10 study-specific RRs ranged from a low of 0.96 (95%CI = 0.94–0.99, *I*^2^ = 0%, *P* for heterogeneity = 0.51) after omitting the study by Biel *et al*.^8^ to a high of 0.98 (95%CI = 0.95–1.01, *I*^2^ = 0%, *P* for heterogeneity = 0.47) after omitting the study of NHS/NHSII by Merritt *et al*.^7^.

## Discussion

Findings of this meta-analysis of 16 epidemiological studies indicated that there was little evidence of a dose-response relationship between total dietary fat intake and EC risk. When investigating the aforementioned associations by different fat source, non-significant results were still observed.

Our findings are inconsistent with a previous meta-analysis of one cohort and 7 case-control studies which suggested that total dietary fat (RR = 1.72, 95%CI = 1.28–2.32) intake was associated with an increased risk of EC^21^. However, these included studies reported the aforementioned results with mixed units of total dietary fat intake, such as grams/day or % calories from fat^21^. For example, Littman *et al*.^23^ reported the association between percent energy from fat which was one of the categories of energy and EC risk on the basis of a population-based case-control study with 679 EC cases and 944 controls. Additionally, Potischman *et al*.^24^ presented the relationship between fat calories and risk of EC in a population-based case-control study. The similar units of dietary fat intake were presented in study of Goodman *et al*.^25^. In contrast, our findings were in accordance with the continuous update project of WCRF/AICR which found no evidence between total dietary fat intake and EC risk. However, the findings had high heterogeneity which limited its interpretation. Furthermore, the previous study failed to carry out the subgroup analysis stratified by the source of dietary fat.

When stratified by study design, we only observed inverse association between total dietary fat intake and EC risk in cohort studies. Such discrepancy could be partly attributed to the methodological differences in study designs. Compared with case-control studies, prospective studies are less susceptible to bias (e.g. recall bias, selection bias) due to their nature. Additionally, on the basis of the updated NOS, less case-control studies fulfilled these criteria than cohort studies. However, since only 4 cohort studies were included, the possibility of chance finding could not be rule out. Therefore, more prospective studies are needed in the future. Similar to the findings of prospective studies, we could not rule out the possibility of the chance findings of the significant results of studies in Europe (n = 4). On the other hand, the difference could also result from the fact that different populations consume different amount of dietary fat. For example, Furberg *et al*.^13^ reported the mean total dietary fat intake of 54.9g/day in 24,460 women, aged 20–49 years, attended a Norwegian health screening. In contrast, McCann *et al*.^16^ reported the mean intake of 76.7 g/day in 639 population-based controls in New York.

This meta-analysis had several strengths. To the best of our knowledge, this is the most update meta-analysis consists of systematical searching and study quality evaluation and low heterogeneity. Additionally, compared with the previous meta-analysis and the continuous update project of WCRF/AICR^21^,^22^, large numbers of EC events and non-cases were included which should have provided sufficient statistical power to detect this putative association. Notably, we also carried out numerous subgroup and sensitivity analyses which suggested the findings were robust. Since the analysis for the highest *versus* lowest category will be strongly influenced by the highest or lowest category of total dietary fat intake of each included studies which were considerable different, we only carried out the dose-response analysis of the aforementioned association in the present study.

Several limitations need to be considered when interpreting our results. First, by its very nature, a meta-analysis inherits all the shortcomings of the constituent studies. Since all included studies were observational study design, the association between total fat intake and EC risk could result from unmeasured or residual confounding by other dietary or lifestyle factors. Higher dietary fat intake is typically associated with other unhealthy behaviors, such as higher intakes of total energy and red meat; obesity; and higher prevalence of cigarette smoking and alcohol drinking. Except for two studies only adjusted age^13^,^17^, the other studies adjusted for these potential confounding factors in their primary analysis, although not all potential confounders were adjusted for in every study. The null associations persisted in almost all subgroups regardless of adjustment potential confounders or important risk factors. Furthermore, the results of meta-regression analyses found no evidence that these findings differed significantly between studies adjusted for these confounders or not. Second, all included studies used food frequency questionnaires to evaluate dietary intake. Since this, measurement errors could be introduced which might obscure the association between dietary fat intake and risk of EC. However, none of these studies reported results corrected for measurement errors. Notably, only the NHS/NHSII mentioned that the dietary information was reassessed every approximately 4 years after baseline until the end of follow-up^7^. Furthermore, as one of the three contributors to the energy source, dietary fat intake was highly correlated with energy intake. Adjustment for total energy intake in the multivariable models should be a major concern. Although the result of meta-regression did not show difference of whether using energy-adjusted models (the residual and nutrient density models), since these two aforementioned models generally have more power to detect associations when the exposure variable is categorized, further studies should carefully address this issue in the future.

In conclusion, our systematic review and meta-analysis of 16 epidemiological studies investigating the relationship of total dietary fat intake with EC indicates that any effect of total dietary fat intake is likely to be small. Further prospective studies are warranted to confirm these findings. Furthermore, a collaborative re-analysis of primary data from the individual studies, after standardizing exposure and developing a uniform approach for confounding control, would be in the position to provide a more definitive answer regarding the dietary fat-EC association.

## Materials and Methods

### Search Strategy

Two independent investigators (RH and Q-JW) systematically searched PubMed (MEDLINE), EMBASE, and Web of Science from each database’s inception to the end of June, 2015 to identify relevant epidemiological studies. The following search keywords were used: (diet OR dietary OR fat OR fatty) AND (endometrium OR endometrial) AND (cancer OR tumor OR carcinoma OR neoplasm). A manual review of references from eligible studies as well as several review articles^21^,^26^ was also performed. This search strategy was similar to previous studies^27^,^28^. We followed the Preferred reporting items for systematic reviews and meta-analyses (PRISMA) guidelines to plan, conduct and report this meta-analysis^29^.

### Study Selection and exclusion

To be included in this analysis, a study must have (i) an observational study design; (ii) evaluated the association between dietary fat intake and EC risk; and (iii) presented RR, odds ratio (OR), or hazard ratio (HR) estimates with 95%CIs or necessary data for calculation^27^. If several publications involved overlapped individuals, we included the study with the most patients.

The studies were excluded by the following exclusion criteria: (i) were randomized controlled trials, reviews without original data, ecological studies, editorials, and case reports; (ii) reported the risk estimates that could not be summarized (such as reported the risk estimates without 95%CIs); and (iii) reported the outcome as EC mortality or recurrence^27^.

### Data extraction and quality assessment

Data were extracted by two investigators (RH and Q-JW) using a data extraction form and entered into a database. All differences were resolved by discussion with the third investigator (LJ). For each included study, we extracted the following information: last name of the first author, publication year, geographic location, number of cases/controls (size of cohort), age at recruitment, mean follow-up year of prospective study, exposure assessment and categories, and study-specific adjusted estimates with their 95% CIs for the highest compared with the lowest category of intake (including adjusted confounders information if applicable). If there were multiple estimates for the association, we used the estimate adjusted for the most appropriate confounding variables, like previous studies^27^,^30^,^31^,^32^.

An update Newcastle-Ottawa Scale (NOS)^27^,^32^,^33^,^34^ uses four quality parameters including selection, comparability, exposure/outcome, and energy-adjusted model was used to assess the methodological quality of all included studies. We evaluated these included studies on the basis of NOS instead of scoring them and categorizing them into high or low quality according to the scores, since quality scoring might not only submerge important information by combining disparate study features into a single score but introduce somewhat arbitrary subjective element into the analysis^35^,^36^,^37^.

### Statistical analysis

As the absolute risk of EC is low and therefore we interpreted all risk estimates as relative risk (RR) for simplicity^27^. For study^7^ reported aforementioned associations on the basis of the EPIC as well as the NHS/NHSII but in one article, we treated it as two included studies. For the NHS/NHSII^7^ provided the cumulative average diet as well as the baseline diet intake, we included the risk estimates of cumulative average diet in the main analyses. Furthermore, Merritt *et al*.^7^ provided the risk estimates of total dietary fat intake on the basis of NHS/NHSII but Cui *et al*.^6^ provided the risk estimates of plant-based fat and animal-based fat intake on the basis of NHS. Therefore, we only include the study of Merritt *et al*.^7^ when calculating the total number of EC cases and non-cases.

To examine the associations between the dietary fat intake and EC risk, the summary RR with 95%CIs were estimated by summarizing the risk estimates of each study using the random effect models, which considered both within- and between-study variation^38^. We summarized the study-specific RR for each 30g/day increment in dietary fat intake. The study-specific trend from the correlated log RR across the categories of dietary fat intake was computed by using the generalized least-squares trend estimation method developed by Greenland and Longnecker^39^ and Orsini *et al*.^40^. For studies reported the risk estimates as per standard deviation (SD) increment of total fat intake, we used previously described methods^41^,^42^ to recalculate risk estimates into per 30g/day increment. Furthermore, a potential nonlinear dose-response relationship between the dietary fat intake and the EC risk was modeled by using restricted cubic splines with three knots at fixed percentiles (10, 50 and 90%) of the distribution of exposure^43^,^44^,^45^,^46^. We calculated the overall *P*-value by testing that these two regression coefficients were simultaneously equal to zero. We calculated a *P*-value for nonlinearity by testing that the coefficient of the second spline was equal to zero. The details of this method has been published elsewhere^47^,^48^.

For conducting the dose-response meta-analysis, the following information were needed: (i) the distribution of cases and non-cases and the risk estimates with the variance estimates for at least three quantitative exposure categories; (ii) the median or mean level of these exposures in each category (if reported by ranges, mean level was calculated by averaging the lower and upper bound; if the lowest category was open ended, the lowest boundary was considered to be zero; if the highest category was open ended, the open-ended interval length was assumed to be the same as the adjacent interval). Given this, 11, 5, and 6 studies met the criteria and were included in the dose-response analysis of total fat, plant-based, and animal-based fat intake and EC risk, respectively.

To investigate the possible sources of heterogeneity of main results, we carried out stratified analyses by the following study features: study design (cohort *versus* case-control studies), type of control subject (population-based *versus* hospital-based), geographic location (North America *versus* Europe), validated food frequency questionnaire (yes *versus* no), number of EOC cases (≥500 versus <500), energy-adjusted model (yes *versus* no), and adjustment for potential confounders including total energy intake, body mass index, cigarette smoking, parity, oral contraceptive use, menopausal status, and hormone replacement therapy use. Heterogeneity between subgroups was evaluated by meta-regression^27^,^32^,^33^,^34^.

Small study bias, such as publication bias can reflect genuine heterogeneity, chance, or other reasons for differences between small and large studies which was evaluated with Egger’s regression asymmetry test^49^. A *P*-value of 0.05 was used to determine whether significant publication bias existed. Furthermore, sensitivity analyses were conducted by deleting each study in turn to reflect the influence of individual data on the overall estimate. All statistical analyses were performed with Stata (version 12; StataCorp, College Station, TX).

## Additional Information

**How to cite this article**: Jiang, L. *et al*. Dietary fat intake and endometrial cancer risk: dose-response meta-analysis of epidemiological studies. *Sci. Rep.* **5**, 16693; doi: 10.1038/srep16693 (2015).

## Figures and Tables

**Table 1:** Characteristics of studies included in the meta-analysis.

First author (ref), year, Country	No. of cases/cohort, age, follow-up	Energy-adjusted model (unit)	Exposure categories (Dietary assessment)	Risk estimates (95% CI)	Matched/adjusted factors
Prospective study					
Merritt *et al*.^7^, 2015, Europe	1303/301,107 (25–70y), 11y	Residual (g/day)	Quartile 4 *vs.* Quartile 1 Total fat Plant fat Animal fat (Validated FFQ)	Hazard ratio 0.84 (0.71–0.99) 1.00 (0.82–1.23) 0.94 (0.80–1.11)	BMI, total energy intake, smoking status, age at menarche, OC use, parity, and a combined variable for menopausal status and postmenopausal hormone use and were stratified by age and study center
Merritt *et al*.^7^, 2015, USA	1531/155,406 (30–55y), 25y	Residual (g/day)	Quartile 4 *vs.* Quartile 1 Total fat (Validated FFQ)	Hazard ratio 0.95 (0.81–1.11)	BMI, total energy intake, smoking status, age at menarche, OC use, parity, and menopausal status and postmenopausal hormone use and were stratified by age, cohort, and the 2-year questionnaire cycle
Cui *et al*.^6^, 2011, USA	669/68,070 (30–55y), 21y	N/A (g/day)	Quintile 5 *vs.* Quintile 1 Plant fat Animal fat (Validated FFQ)	Relative Risk 0.84 (0.65–1.08) 0.99 (0.77–1.26)	Age, follow-up period, total energy, smoking, OC use, postmenopausal hormone use, age at menopause, parity, age at menarche, hypertension, diabetes, BMI
Furberg *et al*.^13^, 2003 Norway	130/24,460 (20–49), 15.7y	N/A (g/day)	Per 19.4g/day Total fat (Validated FFQ)	Relative Risk 0.99 (0.82–1.20)	Age
Jain *et al*.^15^, 2000, Canada	221/56,837 (40–59y), 9y	Residual (g/day)	Quartile 4 *vs.* Quartile 1 Total fat Plant fat Animal fat (Validated FFQ)	Relative Risk 0.84 (0.58–1.23) 1.32 (0.90–1.93) 0.60 (0.40–0.90)	Total energy, age, BMI, ever smoked, used OC, used HRT, university education, livebirths, age at menarche
Zheng *et al*.^18^, 1995, USA	216/23,000 (55–69y), 7y	Residual (g/day)	Quintile 5 *vs.* Quintile 1 Plant fat Animal fat (Validated FFQ)	Relative Risk 0.90 (0.50–1.60) 1.00 (0.50–1.80)	Age, age at menopause, post-menopausal hormone use, and total energy intake
Case-control study Biel *et al*.^8^, 2011, Canada, PC-CS	506/981 (mean, 58.7/58.3y)	Residual (g/day)	Quartile 4 *vs.* Quartile 1 Total fat (Validated FFQ)	Odds Ratio 1.12 (0.80–1.55)	Age, total energy intake, age at menarche, BMI, parity, educational level, hypertension history, OC use, HRT use combined with menopausal status, and alcohol consumption
Yeh *et al*.^9^, 2009, USA, HC-CS	541/541 (mean, 63.3/63.2y)	N/A (g/day)	Quartile 4 *vs.* Quartile 1 Total fat (FFQ)	Odds Ratio 1.21 (0.67–2.21)	Age, BMI, exogenous estrogen use, smoking, total menstrual months, total energy, total protein and carbohydrates intake
Lucenteforte *et al*.^10^, 2008, Italy, HC-CS	454/908 (median, 60/61y)	Residual (g/day)	Quintile 5 *vs.* Quintile 1 Total fat (Validated FFQ)	Odds Ratio 1.10 (0.70–1.60)	Age, study centre, year of interview, education, PA, BMI, history of diabetes, age at menarche, age at menopause, parity, OC use, HRT use, total energy intake, according to the residual models
Xu *et al*.^11^, 2007, China, PC-CS	1204/1212 (mean, 54.5/54.6y)	Presented (g/1000 kcal/d)	Quintile 5 *vs.* Quintile 1 Total fat Plant fat Animal fat (Validated FFQ)	Odds Ratio 1.10 (0.90–1.50) 0.60 (0.50–0.80) 1.50 (1.20–2.00)	Age, education, menopausal status, diagnosis of diabetes, alcohol consumption, PA, BMI, and total energy intake
Salazar-Martinez *et al*.^12^, 2005, Mexico, HC-CS	85/629 (mean, 51.7/57.1y)	Residual (g/day)	Tertile 3 *vs.* Tertile 1 Total fat Plant fat Animal fat (Validated FFQ)	Odds Ratio 1.45 (0.61–3.44) 1.50 (0.68–3.32) 1.19 (0.55–2.58)	Age, total energy intake, number of live births, BMI, PA, and diabetes
McCann *et al*.^16^, 2000, USA, PC-CS	232/639 (mean, 63.5/55.9y)	N/A (g/day)	Quartile 4 *vs.* Quartile 1 Total fat (Validated FFQ)	Odds Ratio 1.60 (0.70–3.40)	Age, education, BMI, diabetes, hypertension, pack-years cigarette smoking, age at menarche, parity, OC use, menopause status, postmenopausal estrogen use, and total energy intake
Jain *et al*.^14^, 2000, Canada, PC-CS	552/562 (30–79y)	Residual (g/day)	Quartile 4 *vs.* Quartile 1 Total fat Animal fat (Validated FFQ)	Odds Ratio 1.21 (0.84–1.83) 1.66 (1.15–2.40)	Total energy, age, body weight, ever smoked, history of diabetes, used OC, used HRT, university education, live births, age at menarche
Tzonou *et al*.^17^, 1996, Greece, HC-CS	145/298 (N/A)	N/A (g/day)	Quartile 4 *vs.* Quartile 1 Total fat (FFQ)	Odds Ratio 0.72 (0.42–1.25)	Age
Barbone *et al*.^19^, 1993, USA, HC-CS	168/334 (mean, 64/63y)	Residual (g/day)	Tertile 3 *vs.* Tertile 1 Plant fat Animal fat (FFQ)	Odds Ratio 0.60 (0.30–1.10) 1.30 (0.70–2.60)	Age, race, years of schooling, total calories, use of unopposed estrogens, obesity, shape of obesity, smoking, age at menarche, age at menopause, number of pregnancies, diabetes, and hypertension
Shu *et al*.^20^, 1993, China, PC-CS	268/268 (mean, 56/56.4y)	N/A (g/day)	Quartile 4 *vs.* Quartile 1 Plant fat Animal fat (FFQ)	Odds Ratio 1.20 (0.70–1.90) 3.50 (2.00–6.00)	Age, number of pregnancies, BMI, and animal fat (for plant fat)

BMI, body mass index; CI, confidence interval; HC-CS, hospital-based case-control study; PA, physical activity; PC-CS, population-based case-control study; N/A, not available; OC, oral contraceptive; FFQ, food frequency questionnaire.

^*^Risk estimates were calculated from published data with EpiCalc 2000 software (version 1.02; Brixton Health).

**Table 2:** Methodological quality of prospective studies included in the meta-analysis*.

First author (reference), publication year	Representativeness of the exposed cohort	Selection of the unexposed cohort	Ascertainment of exposure	Outcome of interest not present at start of study	Control for important factor or additional factor†	Assessment of outcome	Follow-up long enough for outcomes to occur‡	Adequacy of follow-up of cohorts§	Using an energy-adjusted model
Merritt *et al*.^7^, 2015	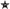	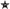	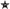	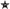	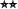	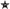	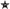	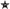	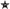
Merritt *et al*.^7^, 2015	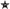	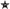	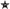	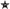	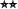	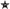	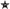	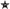	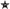
Cui *et al*.^6^, 2011	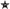	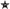	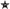	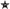	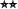	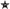	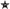	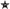	–
Furberg *et al*.^13^, 2003	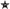	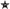	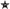	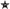	–	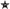	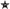	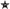	–
Jain *et al*.^15^, 2000	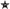	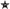	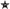	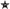	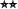	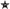	–	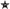	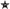
Zheng *et al*.^18^, 1995	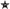	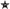	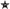	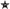	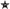	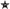	–	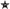	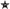

^*^A study could be awarded a maximum of one star for each item except for the item Control for important factor or additional factor. The definition/explanation of each column of the Newcastle-Ottawa Scale is available from (http://www.ohri.ca/programs/clinical_epidemiology/oxford.asp).

^†^A maximum of 2 stars could be awarded for this item. Studies that controlled for total energy intake received one star, whereas studies that controlled for other important confounders such as body mass index, reproductive factors received an additional star.

^‡^A cohort study with a follow-up time >10 y was assigned one star.

^§^A cohort study with a follow-up rate >75% was assigned one star.

**Table 3:** Methodological quality of case-control studies included in the meta-analysis*.

First author (reference), publication year	Adequate definition of cases	Representativeness of cases	Selection of control subjects§	Definition of control subjects	Control for important factor or additional factor†	Exposure assessment	Same method of ascertainment for all subjects	Non response Rate‡	Using an energy-adjusted model
Biel *et al*.^8^, 2011	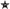	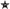	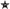	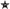	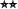	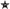	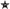	–	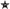
Yeh *et al*.^9^, 2009	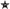	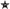	–	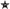	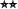	–	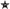	–	–
Lucenteforte *et al*.^10^, 2008	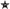	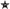	–	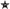	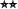	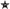	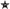	–	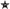
Xu *et al*.^11^, 2007	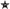	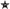	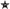	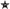	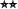	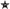	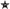	–	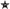
Salazar-Martinez *et al*.^12^, 2005	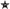	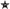	–	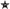	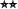	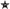	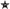	–	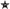
McCann *et al*.^16^, 2000	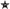	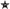	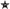	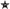	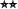	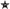	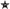	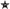	–
Jain *et al*.^14^, 2000	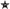	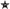	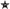	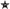	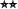	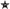	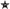	–	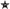
Tzonou *et al*.^17^, 1996	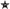	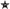	–	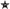	–	–	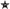	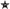	–
Barbone *et al*.^19^, 1993	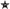	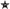	–	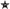	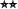	–	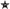	–	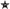
Shu *et al*.^20^, 1993	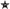	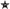	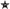	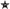	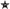	–	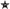	–	–

^*^A study could be awarded a maximum of one star for each item except for the item Control for important factor or additional factor. The definition/explanation of each column of the Newcastle-Ottawa Scale is available from (http://www.ohri.ca/programs/clinical_epidemiology/oxford.asp).

^§^One star was assigned if the control subjects were population-based.

^†^A maximum of 2 stars could be awarded for this item. Studies that controlled for total energy intake received one star, whereas studies that controlled for other important confounders such as body mass index, reproductive factors received an additional star.

^‡^One star was assigned if there was no significant difference in the response rate between control subjects and cases by using the chi-square test (P > 0.05).

**Table 4:** Summary risk estimates of the association between dietary fat intake and endometrial cancer risk, dose-response analysis (per 30 g/day increment).

	No. of study	Summary RR (95%CI)	*I*^2^ value (%)	*P*_h_*	*P*_h_**
Total dietary fat	11	0.97 (0.94–1.001)	0	0.44	
Plant-based fat	5	1.05 (0.94–1.18)	0	0.63	
Animal-based fat	6	1.17 (0.92–1.36)	85.0	<0.01	
Subgroup analyses of total dietary fat					
Study design					0.13
Cohort study	4	0.95 (0.91–0.98)	0	0.85	
Case-control study	7	1.01 (0.96–1.06)	0	0.63	
Type of control subjects					0.30
Population-based	3	1.05 (0.97–1.13)	0	0.95	
Hospital-based	4	0.99 (0.93–1.05)	0	0.41	
Geographic location					0.31
North America	7	0.99 (0.95–1.04)	25.7	0.23	
Europe	4	0.95 (0.91–0.99)	0	0.88	
Validated FFQ					0.61
Yes	10	0.97 (0.94–1.01)	7.6	0.37	
No	1	0.95 (0.87–1.03)	N/A	N/A	
Number of cases					0.67
≥500	5	0.98 (0.94–1.03)	35.5	0.19	
<500	6	0.96 (0.91–1.01)	0	0.61	
Energy-adjusted model					0.51
Yes	7	0.97 (0.93–1.01)	15.6	0.31	
No	4	0.99 (0.93–1.06)	0	0.50	
Adjustment for potential confounders					
Total energy intake					0.63
Yes	9	0.98 (0.94–1.01)	17.8	0.28	
No	2	0.95 (0.88–1.03)	0	0.84	
Body mass index					0.63
Yes	9	0.98 (0.94–1.01)	17.8	0.28	
No	2	0.95 (0.88–1.03)	0	0.84	
Cigarette smoking					0.94
Yes	7	0.98 (0.94–1.02)	28.6	0.21	
No	4	0.97 (0.91–1.04)	0	0.67	
Parity					0.63
Yes	9	0.98 (0.94–1.01)	17.8	0.28	
No	2	0.95 (0.88–1.03)	0	0.84	
Oral contraceptive use					0.62
Yes	7	0.97 (0.93–1.00)	12.1	0.34	
No	4	0.99 (0.92–1.06)	0	0.42	
Menopausal status					0.70
Yes	6	0.97 (0.94–1.01)	7.7	0.37	
No	5	0.96 (0.91–1.02)	9.3	0.35	
Hormone replacement therapy use					0.86
Yes	8	0.98 (0.94–1.01)	18.5	0.28	
No	3	0.96 (0.89–1.04)	0	0.51	

CI, confidence interval; N/A, not available; RR, relative risk.

^*^P-value for heterogeneity within each subgroup.

^**^P-value for heterogeneity between subgroups with meta-regression analysis in random-effect model.
